# Slit-Robo signaling supports motor neuron avoidance of the spinal cord midline through DCC antagonism and other mechanisms

**DOI:** 10.3389/fcell.2025.1563403

**Published:** 2025-04-10

**Authors:** Kelsey R. Nickerson, Ferass M. Sammoura, Yonghong Zhou, Alexander Jaworski

**Affiliations:** ^1^ Department of Neuroscience, Brown University, Providence, RI, United States; ^2^ Robert J. and Nancy D. Carney Institute for Brain Science, Brown University, Providence, RI, United States

**Keywords:** axon guidance, neuronal migration, spinal cord, motor neuron, robo signaling, crosstalk, floor plate

## Abstract

Axon pathfinding and neuronal migration are orchestrated by attractive and repulsive guidance cues. In the mouse spinal cord, repulsion from Slit proteins through Robo family receptors and attraction to Netrin-1, mediated by the receptor DCC, control many aspects of neural circuit formation. This includes motor neuron wiring, where Robos help prevent both motor neuron cell bodies and axons from aberrantly crossing the spinal cord midline. These functions had been ascribed to Robo signaling being required to counter DCC-mediated attraction to Netrin-1 at the midline, either by mediating repulsion from midline-derived Slits or by silencing DCC signaling. However, the role of DCC in promoting motor neuron and axon midline crossing had not been directly tested. Here, we used *in vivo* mouse genetics and *in vitro* axon turning assays to further explore the interplay between Slit and Netrin signaling in motor neuron migration and axon guidance relative to the midline. We find that DCC is a major driver of midline crossing by motor axons, but not motor neuron cell bodies, when *Robo1* and *Robo2* are knocked out. Further, *in vitro* results indicate that Netrin-1 attracts motor axons and that Slits can modulate the chemotropic response to Netrin-1, converting it from attraction to repulsion. Our findings indicate that Robo signaling allows both motor neuron cell bodies and axons to avoid the midline, but that only motor axons require this pathway to antagonize DCC-dependent midline attraction, which likely involves a combination of mediating Slit repulsion and directly influencing Netrin-DCC signaling output.

## Introduction

Assembly of neural circuits during embryonic development requires the guidance of nascent axons to their correct targets. This process of axon pathfinding is instructed by molecular cues that signal through receptors on the leading process of the axon, the growth cone (Kolodkin and Tessier-Lavigne, 2011). While axon guidance cues are often categorized as either attractants or repellants, some of them can exert both attractive or repulsive effects, depending on context. A classic example is the secreted protein Netrin-1, which can signal attraction through the receptor Deleted in Colorectal Cancer (DCC) and repulsion via Unc5 family members (Keino-Masu et al., 1996; Leonardo et al., 1997). While the complement of available Netrin receptors is a key determinant of a neuron’s response to this cue, the level of cAMP in the growth cone also influences the valence of Netrin-1’s effects on axon extension (Song et al., 1998), and extracellular signals, such as laminin, can modulate the intracellular cAMP concentration to switch Netrin-mediated attraction to repulsion (Höpker et al., 1999). Hence, multiple intrinsic and extrinsic factors dictate how an axon will respond to a given cue. This concept extends to cross-regulatory interactions between guidance cues. Growing axons *in vivo* usually integrate information from several cues that either collaborate to steer a growth cone in the same direction or exert opposite effects on axon extension. While simple summation of the attractive and repulsive effects of multiple ligands through parallel signaling pathways is observed in some cases, signal crosstalk can drive synergistic, permissive, or hierarchical integration of guidance information (Morales and Kania, 2017). Netrin-1 repulsion, for instance, synergizes with ephrin-B2 repulsion in motor axon pathway choice in the developing vertebrate limb (Poliak et al., 2015), and motor axon attraction to Netrin-1 in the spinal cord has been proposed to be silenced by axon repellants of the Slit family through hierarchical receptor interactions (Bai et al., 2011). The contexts in which different mechanisms of guidance cue integration drive axon pathfinding *in vivo* have not been fully delineated.

Netrin-mediated attraction and Slit-dependent repulsion control axonal crossing of the nervous system midline in bilaterians, including nematode worms, flies, mice, and humans (Dickson and Zou, 2010). In the mouse spinal cord and hindbrain, floor plate cells at the ventral midline secrete both Netrin-1 and all three Slit paralogs – Slit1, Slit2, and Slit3 (Kennedy et al., 1994; Brose et al., 1999). Netrin-1 is also produced by radial glia and deposited at the pial surface, and the combined attractive and growth-promoting effects of floor plate- and radial glia-derived Netrin-1 guide commissural axons towards and across the ventral midline (Serafini et al., 1994; Serafini et al., 1996; Dominici et al., 2017; Varadarajan et al., 2017; Moreno-Bravo et al., 2019; Wu et al., 2019). Slit proteins signal axon repulsion through receptors of the Robo family (Blockus and Chedotal, 2016), and floor plate-derived Slits help expel commissural axons from the midline after crossing and prohibit their re-crossing (Zou et al., 2000; Long et al., 2004) while also preventing ipsilaterally projecting neurons from sending axons across the midline in the first place (Farmer et al., 2008). How spinal cord neurons integrate signaling from Netrin-1 and Slits remains incompletely understood.

Motor neurons in the spinal cord and hindbrain project axons towards their muscle targets in the body periphery, and they express both Netrin and Slit receptors during development (Bonanomi and Pfaff, 2010). Limb-innervating motor neurons belonging to the lateral motor column (LMC) use Netrin-1 expressed in the limb mesenchyme to select the correct dorso-ventral axon trajectory; this involves DCC-mediated attraction of motor axons originating from the lateral subdivision of the LMC and Unc5c-dependent repulsion of medial LMC axons (Poliak et al., 2015). Netrin-1 and DCC also regulate earlier aspects of motor neuron development, such as the dorso-ventral positioning of motor neuron cell bodies and of the motor exit points (MEPs) where motor axons leave the central nervous system; here, Netrin-mediated attraction to the midline and Slit-mediated repulsion appear to balance each other, as genetic disruption of either of these signaling pathways has opposing effects on motor neuron and MEP positioning (Kim et al., 2015; Kim et al., 2017). In mice lacking the Slit receptors Robo1 and Robo2, motor neuron cell bodies and axons can even be observed entering the ventral midline, which they usually avoid (Bai et al., 2011; Kim et al., 2015; Kim et al., 2017; Gruner et al., 2019). In one study, ectopic motor axon midline crossing in *Robo1* ^*−/−*^ *; Robo2* ^*−/−*^ (*Robo1/2* ^*−/−*^) double knockout mice was attributed to a gain of Netrin-mediated midline attraction rather than a loss of Slit-mediated midline repulsion, invoking a hierarchical crosstalk model where Slit signaling through Robos suppresses attraction via Netrin-DCC (Bai et al., 2011). However, this model has not been validated by phenotypic rescue of motor neuron cell body and axon crossing of the midline via inactivation of DCC; other, Slit/Robo-independent DCC silencing mechanisms have been identified (Bonanomi et al., 2019); and different studies also report conflicting findings regarding the baseline ability of motor neurons to respond to Netrin-1 *in vitro* (Varela-Echavarría et al., 1997; Bai et al., 2011; Poliak et al., 2015; Kim et al., 2017). The precise roles, and interplay, of the Netrin-DCC and Slit-Robo pathways in motor neuron migration and axon guidance relative to the spinal cord midline have therefore remained somewhat enigmatic.

Here, we combined mouse genetics and *in vitro* axon guidance assays to revisit the functions of Netrin-DCC and Slit-Robo signaling in spinal motor neurons. Our results indicate that aberrant midline crossing by motor neuron cell bodies and axons in mice lacking Robo1 and Robo2 are not interdependent and that DCC contributes to axon, but not cell body entry into the ventral commissure. Further, we find that motor axons are attracted by Netrin-1 and that Slits can convert this attractive effect to repulsion. These results support a hierarchical relationship between Slit-Robo and Netrin-DCC signaling in motor axon guidance that goes beyond a silencing interaction and involves a Slit-induced change in the valence of axonal responses to Netrin-1.

## Materials and methods

### Animals

All experimental procedures had institutional approval through Brown University’s Institutional Animal Care and Use Committee (current protocol number 24-11-0002) and followed the guidelines provided by the National Institutes of Health. Null alleles for *Robo1* (Long et al., 2004), *Robo2* (Grieshammer et al., 2004), and *DCC* (Fazeli et al., 1997) have been described before, and mice carrying these mutations were genotyped by PCR as originally reported. Mice were maintained on a CD-1 background. *Robo1* ^*+/−*^ *; Robo2* ^*+/−*^ *; DCC* ^*+/−*^ triple heterozygous animals were generated by crossing mice carrying the closely linked *Robo1* and *Robo2* knockout alleles (Chen et al., 2008) to *DCC* ^*+/−*^ mice, and experimental litters for phenotype analysis were generated by intercrossing of triple heterozygotes. For timed pregnancies, the day of vaginal plug was defined as embryonic day (E) 0.5, and littermate embryos of either sex were used for all experiments.

### Immunohistochemistry

All spinal cord transverse cryosections were collected from brachial level (i.e., cervical and upper thoracic spinal cord segments with visible limb buds in the same sections). Immunohistochemistry (IHC) on 20-μm-thick cryosections was performed as previously described (Jaworski et al., 2010). Antibody labeling of neuronal cultures following live imaging in Dunn chambers was performed essentially as reported before (Pak et al., 2020). Primary antibodies used for IHC were rabbit polyclonal antibodies against class III β-tubulin (TuJ1) (Biolegend, 1:500) (Hu et al., 2006), Peripherin (Prph) (Millipore, 1:200) (Xiao et al., 2008), and FoxP1 (Abcam, 1:500) (Sheng et al., 2019), and mouse monoclonal antibodies against neurofilament (NF) (DSHB, 1:200) (Dodd et al., 1988) and Islet (Isl) 1/2 (DSHB, 1:200) (Tsuchida et al., 1994). Secondary antibodies (all from Invitrogen; 1:200) were Alexa488-conjugated donkey anti-rabbit, Alexa594-conjugated donkey anti-rabbit, Alexa488-conjugated donkey anti-mouse, and Alexa594-conjugated donkey anti-mouse. Hoechst 33342 (Molecular Probes, 1:1,000) was added with the secondary antibodies. Images were acquired on a Nikon Ti-E microscope.

### Dunn chamber axon turning assay

Dunn chamber axon turning assays were adapted for motor neurons but essentially performed as previously described for spinal commissural neurons (Pak et al., 2020), with few modifications. E10.5 ventral spinal cord was dissected and dissociated as previously described (Suter et al., 2020), pooling tissue from multiple embryos. Cells were plated on nitric acid-washed and baked 18-mm coverslips coated with 100 μg/mL PDL and 5 μg/mL laminin, cultured in motor neuron media [1x penicillin/streptomycin/glutamine, 2% B-27 (both Gibco), 0.5% glucose, 10 ng/mL BDNF (Cell Sciences), 10 ng/mL NT-3 (Sigma) in Neurobasal-A medium (Gibco)], and used for experiments 16–26 hours (h) after plating. The age of neurons at the time of the experiment was therefore E10.5 + 1 day *in vitro* (DIV). Media from pre-culturing of neurons was reused in Dunn chambers, recombinant mNetrin-1 or mSlit2-N (both Biotechne/R&D Systems) was added at indicated concentrations to media in the Dunn chamber outer well, and ≈30–40 visual fields [containing 8–20 analyzable motor neurons per experimental replicate (n)] covering the bridge region of each chamber were imaged repeatedly over 2 h. For studying effects of mSlit2-N bath application on axon turning in response to mNetrin-1, mSlit2-N was added at 1 μg/mL to media used for Dunn chambers (inner and outer well), while 250 ng/mL mNetrin-1 was added to the outer well only. Images were acquired on a Nikon Ti-E microscope.

### Quantification and statistical analysis

#### Quantification of motor neurons entering the midline

To count mispositioned motor neuron cell bodies at the spinal cord midline, brachial spinal cord sections were immunolabeled for Isl1/2 and Tuj1. The number of Isl1/2-positive (Isl1/2^+^) cells in the ventral midline, defined by the area enclosed by the Tuj1^+^ commissural axon bundle and the ventral edge of the central canal, from 6 to 15 sections per animal was quantified and normalized to the total number of sections per animal. Means across multiple animals of the same age and genotype (n = 3–5 animals) were calculated and used for statistical comparison after confirming normal distribution of the data. Statistical significance across multiple groups was assessed using a one-way ANOVA with post-hoc Holm’s test for multiple comparisons (α = 0.05). Pairwise comparisons between groups were analyzed using two-tailed unpaired t-test (*p* = 0.05). To determine the molecular identity of motor neurons in the midline, E10.5 *Robo1/2* ^*−/−*^ brachial spinal cord sections were immunolabeled for Isl1/2 and the LMC-specific transcription factor FoxP1. The number of medial motor column (MMC; Isl1/2^+^/FoxP1^−^) and LMC (Isl1/2^+^/FoxP1^+^) cells in the ventral midline from 3–8 sections per animal (n = 3 animals) was quantified, normalized to the total number of sections per animal, and expressed as mean per animal. All motor neuron counts were performed blinded to animal identities.

#### Quantification of total motor neurons

To count total motor neurons, brachial spinal cord sections were immunolabeled for Isl1/2 and Tuj1. Isl1/2^+^ cells in the ventral horn were counted and expressed as neurons per hemisection, averaging 6–16 hemisections per animal. Means across multiple animals of the same age and genotype (n = 5–9 animals) were calculated and used for statistical comparison after confirming normal distribution of the data. Pairwise comparison between groups was performed using a two-tailed unpaired t-test (*p* = 0.05).

#### Quantification of motor axons crossing the ventral midline

To count motor axons in the ventral midline, brachial spinal cord sections were immunolabeled for Prph and NF. The number of Prph^+^ axons entering the midline from 5 to 15 sections per animal was quantified and normalized to the total number of sections per animal. Means across multiple animals of the same age and genotype (n = 4–9 animals) were calculated and used for statistical comparison after confirming normal distribution of the data. Statistical significance across multiple groups was assessed using a one-way ANOVA with post-hoc Holm’s test for multiple comparisons (α = 0.05). Pairwise comparisons between groups were analyzed using two-tailed unpaired t-test (*p* = 0.05). Analyses were performed blinded to animal identities.

#### Quantification of axon turning in dunn chambers

Quantitative analysis of axon turning in Dunn chambers was performed as described previously (Pak et al., 2020). All analyses were performed blinded to experimental conditions. Motor neurons were identified by post-hoc immunostaining of the imaged coverslip for Isl1/2. For each experimental replicate, axon turning angles from all analyzable motor neurons were averaged, and means across multiple replicates per condition were analyzed for statistical significance using a one-way ANOVA with post-hoc Holm’s test for multiple comparisons (α = 0.05) (n and *p* are indicated in figure legends) after confirming normal distribution of the data.

## Results

### Robo1 and Robo2 prevent MMC motor neurons from entering the spinal cord midline

**FIGURE 1:**
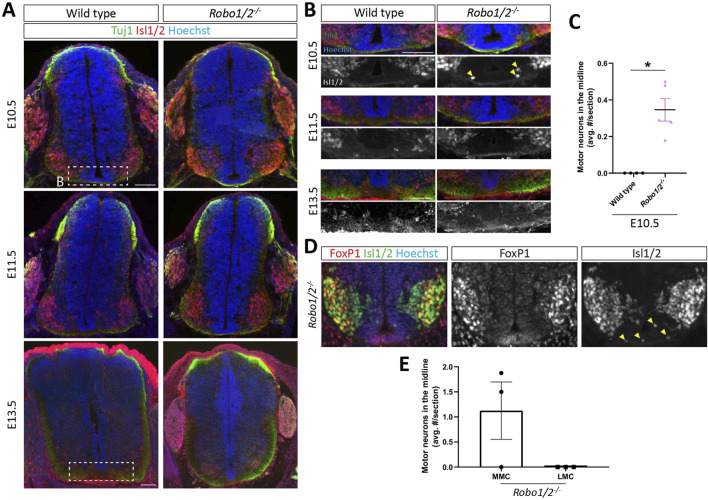
Motor neuron entry into the midline of *Robo1/2* ^*−/−*^ mice. **(A)** Transverse spinal cord sections of E10.5, E11.5, and E13.5 wild-type and *Robo1/2* ^*−/−*^ mice, stained for Isl1/2 and Tuj1. White boxes indicate regions shown in **(B)**. **(B)** Higher magnification views of E10.5, E11.5, and E13.5 wild-type and *Robo1/2* ^*−/−*^ mice. Isolated greyscale channel shows Isl1/2 staining. Yellow arrowheads indicate motor neurons infiltrating the midline. **(C)** The average number of Isl1/2^+^ cells in the midline was quantified in E10.5 wild-type and *Robo1/2* ^*−/−*^ mice. Motor neurons enter the midline in E10.5 *Robo1/2* ^*−/−*^ mice, which is not observed in wild-type sections (n = 4-5 animals/group, *p* = 0.0159). **(D)** Spinal cord sections from E10.5 Robo1/2^−/−^ mice, stained for Foxp1 and Isl1/2. Isolated Foxp1 and Isl1/2 channels are also shown in greyscale. Motor neurons infiltrating the midline stained exclusively for Isl1/2. **(E)** The number of motor neurons in the midline belonging to the MMC (Isl1/2^+^/FoxP1^−^) and LMC (Isl1/2^+^/FoxP1^+^) was quantified (n = 3 animals). Data are represented as means ± SEM. Scale bar for E10.5 and E11.5 sections = 100 µm. Scale bar for E13.5 sections = 100 µm.

Previous studies have reported that hindbrain and spinal cord motor neurons aberrantly migrate into the nervous system midline in mice lacking Robo1 and Robo2 (Kim et al., 2015; Gruner et al., 2019). In the spinal cord, this phenotype had been observed at E9.5 and E10.5, but not E12.5, and the columnar origin of the mispositioned motor neurons had not been determined (Kim et al., 2015). We sought to recapitulate this defect and examine its dependence on DCC-mediated midline attraction. First, we performed IHC using an antibody against the transcription factors Isl1 and Isl2, which mark motor neurons in the ventral spinal cord, and the panaxonal marker TuJ1 on transverse sections of brachial spinal cord from E10.5, E11.5, and E13.5 *Robo1/2* ^*−/−*^ mice and their wild-type littermates. At E10.5 and E11.5, motor neurons still migrate into the ventral horn, and their axons extend into the periphery, while motor neuron generation has ceased by E13.5, and motor axons start innervating their targets (Shirasaki et al., 2006; Wang et al., 2011). Across all ages in both wild-type and *Robo1/2* knockout embryos, most Isl1/2^+^ motor neurons occupy the spinal cord ventral horn, but in E10.5 *Robo1/2* double mutants, a small number of motor neurons is mispositioned within the floor plate area at the ventral midline, which is never observed in wild type (Figures 1A–C). We examined this phenotype more closely by co-labeling with antibodies against the LMC-specific marker FoxP1. We found that, in the spinal cord ventral horn of *Robo1/2* mutant mice, MMC (Isl1/2^+^/FoxP1^−^) and LMC (Isl1/2^+^/FoxP1^+^) motor neurons are spatially segregated as they are in wild type, and that motor neurons in the midline of *Robo1/2* ^*−/−*^ mice are exclusively of MMC, not LMC, identity (Figures 1D, E). The total number of motor neurons in E10.5 spinal cord is comparable between the two genotypes (Supplementary Figure S1), and Isl1/2^+^ neurons are excluded from the midline in wild-type and *Robo1/2* double knockout mice at E11.5 and E13.5 (Figure 1A; Supplementary Figure S2). Thus, Robo1 and Robo2 prevent a subset of MMC motor neurons from migrating into the spinal cord ventral midline, without controlling overall motor neuron number or columnar organization, and aberrantly positioned motor neurons in the midline of *Robo1/2* double knockout mice do not persist past E10.5.

### DCC is not required for motor neuron entry into the midline of *Robo1/2* mutant mice

**FIGURE 2:**
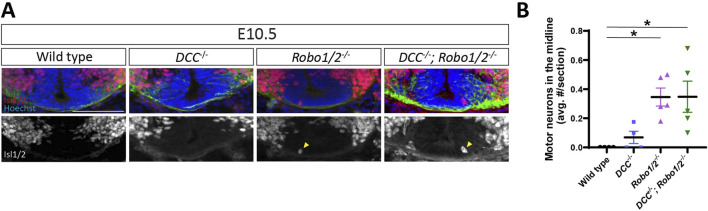
Mispositioning of motor neurons in *Robo1/2* ^*−/−*^ mice is not DCC-dependent. **(A)** Transverse sections of E10.5 wild-type, *DCC* ^*−/−*^, *Robo1/2* ^*−/−*^, and *DCC* ^*−/−*^ *; Robo1/2* ^*−/−*^ mice, stained for Isl1/2 and Tuj1. Isolated Isl1/2 channel is shown in greyscale. Yellow arrowheads indicate Isl1/2^+^ motor neurons in the midline. **(B)** Quantification of motor neurons in the midline of E10.5 wild-type, *DCC* ^*−/−*^, *Robo1/2* ^*−/−*^, and *DCC* ^*−/−*^ *; Robo1/2* ^*−/−*^ mice shows no significant differences in the number of mispositioned motor neurons between wild-type and *DCC* ^*−/−*^ mice (n = 4 animals/group, *p* = 0.7842). However, both *Robo1/2* ^*−/−*^ and *DCC* ^*−/−*^ *; Robo1/2* ^*−/−*^ mice have significantly more mispositioned motor neurons in the midline compared to wild type (n = 4-5 animals/group, *p* = 0.0261 and *p* = 0.0261, respectively). No difference is observed in the average number of motor neurons in the midline between *Robo1/2* ^*−/−*^ and *DCC* ^*−/−*^ *; Robo1/2* ^*−/−*^ groups (n = 5, *p* = 0.9838). Data are represented as means ± SEM. Scale bar = 100 µm.

Netrin-DCC signaling contributes to the ventral positioning of motor neuron cell bodies in the spinal cord (Kim et al., 2015), and the balance between DCC-mediated attraction to floor plate-derived Netrin and Robo1/2-mediated repulsion from midline Slits has been implicated in specifying the dorso-ventral position of MEPs where motor axons emerge from the spinal cord (Kim et al., 2017). To determine whether unbalanced DCC-mediated floor plate attraction causes motor neuron migration into the midline of *Robo1/2* knockout mice, we analyzed triple mutant mice lacking DCC, Robo1, and Robo2, as well as their wild-type, *DCC* ^*−/−*^, and *Robo1/2* ^*−/−*^ littermates. We found that motor neurons aberrantly enter the floor plate region in E10.5 *DCC* ^*−/−*^ *; Robo1/2* ^*−/−*^ embryos, just as they do in *Robo1/2* ^*−/−*^ mice, and they are largely excluded from the midline in *DCC* ^*−/−*^ and wild-type littermates (Figure 2A). Motor neurons are never observed in the midline at E11.5 and E13.5, irrespective of genotype (Supplementary Figure S2). Quantification revealed that loss of DCC does not significantly change the number of mispositioned motor neurons in the *Robo1/2* mutant background (Figure 2B). These results indicate that midline entry of motor neurons in the absence of Robo1 and Robo2 is not driven by DCC signaling.

### Robo1 and Robo2 inhibit motor axon crossing of the ventral commissure

**FIGURE 3:**
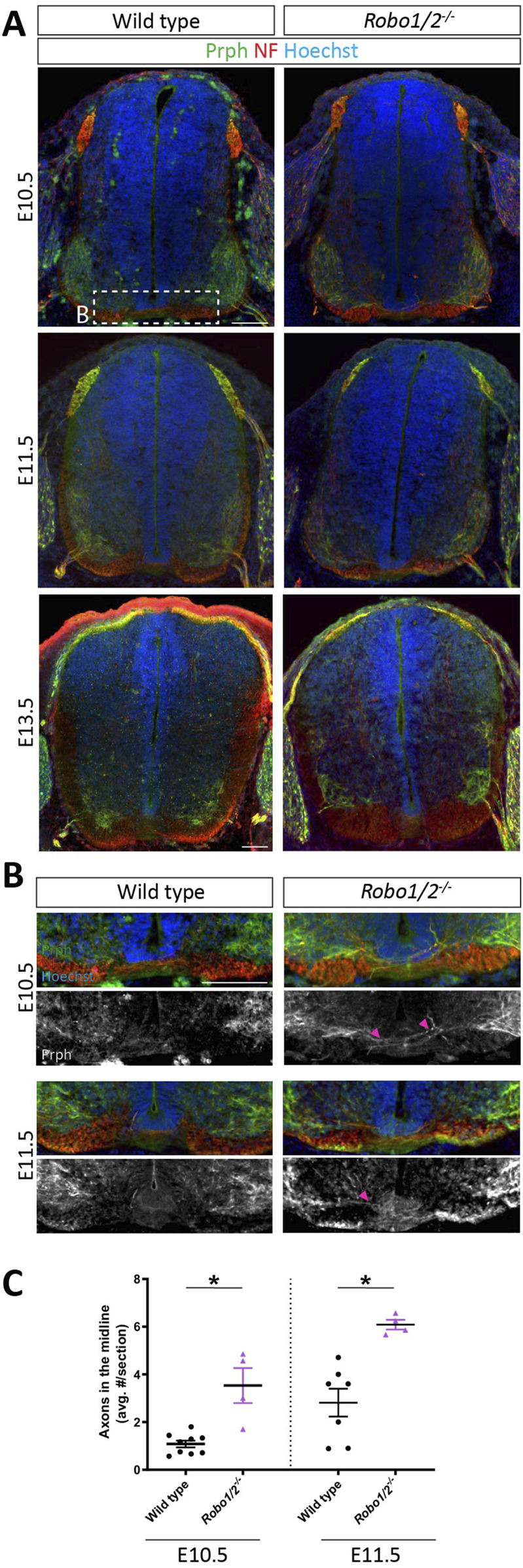
Motor axons aberrantly cross the midline in *Robo1/2* ^*−/−*^ mice. **(A)** Transverse E10.5, E11.5, and E13.5 wild-type and *Robo1/2* ^*−/−*^ mouse spinal cord sections, stained for NF and Prph. **(B)** Higher magnification views of E10.5 and E11.5 wild-type and *Robo1/2* ^*−/−*^ mouse spinal cord sections. Isolated greyscale channel shows Prph staining. Magenta arrowheads indicate Prph^+^ axons entering the midline. **(C)** Quantification of Prph^+^ axons crossing the midline of E10.5 and E11.5 wild-type and *Robo1/2* ^*−/−*^ mice shows significantly increased axon midline crossing in mutants compared to wild type at both ages (E10.5, *p* = 0.0056; E11.5, *p* = 0.0030). Data are represented as means ± SEM (n = 4–9 animals/group). Scale bar for E10.5 and E11.5 sections = 100 µm. Scale bar for E13.5 sections = 100 µm.

Previous reports indicate that motor axons aberrantly project across the ventral midline of the hindbrain and spinal cord in mice lacking Robo1 and Robo2 (Bai et al., 2011; Kim et al., 2017; Gruner et al., 2019), and loss of Robo-dependent silencing of DCC-mediated floor plate attraction had been invoked as a driver of this phenotype (Bai et al., 2011). Because the timecourse of motor axon growth through the ventral commissure in *Robo1/2* ^*−/−*^ mice and its relationship to motor neuron cell body migration into the midline had not been characterized, we first examined motor neuron projections in E10.5, E11.5, and E13.5 *Robo1/2* double knockout embryos and their wild-type littermates. To this end, we stained transverse sections of brachial spinal cord with antibodies against the type III intermediate filament protein Prph, which labels axons in the peripheral nervous system, including motor axons. We found that, at E10.5, motor axons rarely enter the ventral commissure in wild-type embryos, but they are frequently observed in the midline of mice lacking Robo1 and Robo2 (Figures 3A–C); at E11.5, the incidence of motor axons in the commissure has increased in both genotypes, but it is still significantly elevated by about 2-fold in *Robo1/2* ^*−/−*^ embryos when compared to wild type (Figures 3A–C). Hence, ectopic motor axon midline crossing in *Robo1/2* knockout embryos persists longer than motor neuron cell body invasion of the commissure. By E13.5, the number of midline-crossing motor axons has returned to low, comparable levels in both wild-type and *Robo1/2* mutant animals (Figure 3A; Supplementary Figure S3). These results indicate that Robo1 and Robo2 temporarily suppress the tendency of motor axons to cross the midline, although a small number of motor neurons transiently project their axons into the ventral commissure even during normal development in wild-type embryos.

### DCC accelerates motor axon midline crossing in *Robo1/2* mutant mice

**FIGURE 4:**
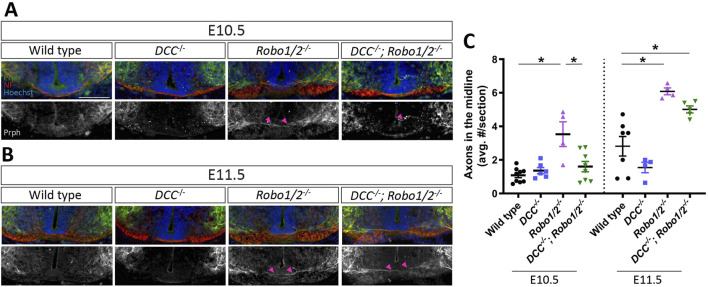
DCC mediates motor axon midline crossing in *Robo1/2* ^*−/−*^ mice at E10.5, but not E11.5. **(A, B)** Transverse E10.5 **(A)** and E11.5 **(B)** wild-type, *DCC* ^*−/−*^, *Robo1/2* ^*−/−*^, and *DCC* ^*−/−*^ *; Robo1/2* ^*−/−*^ embryo sections, stained for NF and Prph. Isolated Prph channel is shown in greyscale. Magenta arrowheads indicate motor axons crossing into the midline. **(C)** Quantification of Prph^+^ axons crossing the midline of E10.5 *Robo1/2* ^*−/−*^ mice shows that aberrant midline crossing is significantly higher compared to wild-type (n = 4–8 animals/group, *p* = 0.0002) and *DCC* ^*−/−*^; *Robo1/2* ^*−/−*^ mice (n = 4–8 animals/group, *p* = 0.0022). E11.5 *Robo1/2* ^*−/−*^ mice also have higher numbers of midline-crossing motor axons compared to wild-type (n = 4–7 animals/group, *p* = 0.0005), but not to *DCC* ^*−/−*^; *Robo1/2* ^*−/−*^ mice (n = 4-5 animals/group, *p* = 0.1366). Additionally, E11.5 *DCC* ^*−/−*^; *Robo1/2* ^*−/−*^ mice have significantly more midline-crossing motor axons compared to wild type (n = 5–7 animals/group, *p* = 0.0063). Data are represented as means ± SEM. Scale bar = 100 µm.

Entry of motor axons into the ventral commissure of *Robo1/2* double knockout mice could be caused by DCC-mediated attraction to floor plate-derived Netrin-1, which is normally either balanced by Robo-mediated repulsion from midline Slits or directly silenced by Robo-DCC inhibitory crosstalk (Bai et al., 2011; Kim et al., 2017). Alternatively, loss of Robo-dependent midline repulsion alone might explain the *Robo1/2* knockout motor axon phenotype without a contribution of DCC signaling, as is the case for motor neuron cell body migration into the midline. To distinguish between these possibilities, we analyzed motor axon crossing of the ventral commissure in *DCC* ^*−/−*^ *; Robo1/2* ^*−/−*^ embryos and their wild-type, *DCC* ^*−/−*^, and *Robo1/2* ^*−/−*^ littermates. We found that, at both E10.5 and E11.5, the number of midline-crossing motor axons in *DCC* ^*−/−*^ mice is similar to wild type (Figures 4A–C), indicating that DCC is not required for the low level of axon midline entry observed in wild-type embryos. The amount of motor axon midline crossing in *DCC* ^*−/−*^ *; Robo1/2* ^*−/−*^ mice at E10.5 is significantly reduced when compared to *Robo1/2* double knockouts, and it is indistinguishable from wild-type and *DCC* ^*−/−*^ mice (Figures 4A, C), indicating a full phenotypic rescue. Thus, DCC is required for aberrant motor axon midline entry in *Robo1/2* mutant mice at E10.5. At E11.5, however, the severity of the phenotype in mice lacking DCC, Robo1, and Robo2 is similar to *Robo1/2* mutant mice (Figures 4B, C), and, at E13.5, the number of motor axons projecting through the ventral commissure is comparable across all genotypes (Figure 4C; Supplementary Figure S3). Hence, DCC is a major driver of early, but not late, motor axon midline crossing in *Robo1/2* knockout mice. Together, these results indicate that DCC signaling accelerates motor axon midline entry in the absence of Robo1 and Robo2, but it is not strictly required for motor axons to cross the commissure.

### Slit2 converts Netrin-1-mediated motor axon attraction to repulsion

**FIGURE 5:**
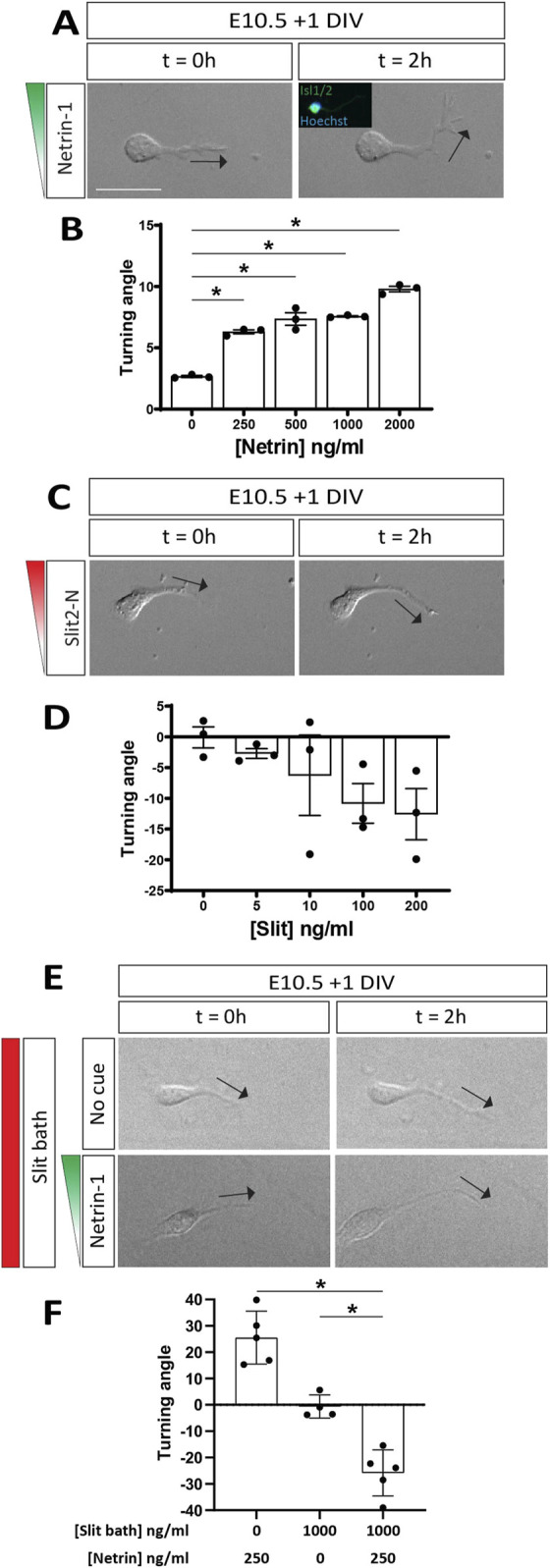
Slit2 converts motor axon attraction by Netrin-1 to repulsion. **(A)** DIC images of E10.5 + 1 DIV motor neurons exposed to a Netrin-1 gradient (250 ng/mL) in a Dunn chamber (t = 0 and 2 h). Direction of axon tip at 0 and 2 h indicated by black arrows. Post-hoc immunofluorescent staining for Isl1/2 confirmed molecular identity of analyzed neurons (inset). Axons turn towards the Netrin-1 gradient. **(B)** Quantification of axon turning angles in response to Netrin-1 gradient shows positive turning angles, indicating attraction, at all tested concentrations of Netrin-1 (n = 3 independent experiments, comparison to control: 250 ng/mL, *p* < 0.0001; 500 ng/mL, *p* < 0.0001; 1,000 ng/mL, *p* < 0.0001; 2000 ng/mL, *p* < 0.0001). **(C)** DIC images of E10.5 + 1 DIV motor neurons exposed to a Slit2-N gradient (100 ng/mL) in a Dunn chamber (t = 0 and 2 h). **(D)** Quantification of turning angles in response to Slit-2N gradients shows negative turning angles, indicating repulsion, albeit not statistically significant (n = 3 independent experiments, comparison to control: 5 ng/mL, *p* = 0.6407; 10 ng/mL, *p* = 0.4848; 100 ng/mL *p* = 0.2130; 200 ng/mL, *p* = 0.1662). **(E)** DIC images of E10.5 + 1 DIV motor neurons exposed to no cue (top panels) or a 250 ng/mL Netrin-1 gradient (bottom panels) with simultaneous bath application of Slit2-N (1,000 ng/mL) in a Dunn chamber (t = 0 and 2 h). Motor axons are repelled by Netrin-1 in the presence of Slit2-N. **(F)** Motor axons exposed to the Netrin-1 gradient in the presence of Slit2-N are strongly repelled, as opposed to the attraction observed in Netrin-1 gradient alone (n = 5 individual experiments, *p* < 0.0001). Additionally, motor axons exposed to Slit-2N (1,000 ng/mL) by bath application alone do not experience attraction or repulsion (n = 4-5 individual experiments; comparison against Netrin-1 gradient and Slit2-N bath: *p* = 0.0014). Data are represented as means ± SEM. Scale bar = 50 µm.

The phenotypic rescue of aberrant motor axon midline crossing by loss of DCC in *Robo1/2* knockout mice, albeit transient, reveals an antagonistic relationship between Netrin-DCC and Slit-Robo signaling in motor axon guidance relative to the midline. This could mean that Netrin-mediated attraction and Slit-dependent repulsion by the floor plate act in parallel, with motor axons passively integrating these opposing signals, or it could indicate that Slits actively suppress motor axon attraction to Netrin via hierarchical crosstalk between the signaling pathways, as previously proposed (Bai et al., 2011). To directly study the effects of these guidance cues and their possible crosstalk on motor axons, we examined the responses of E10.5 motor neurons to gradients of Netrin-1 and Slit2 by live imaging in Dunn chamber axon turning assays (Yam et al., 2009). We used the N-terminal, Robo-binding fragment of Slit2 (Nguyen Ba-Charvet et al., 2001) for these experiments, as Slit2 is prominently expressed by both floor plate and motor neurons at the time when Robo1 and Robo2 prevent ectopic motor axon midline crossing (Brose et al., 1999; Jaworski and Tessier-Lavigne, 2012). First, we established dose-response relationships for each cue. We found that Netrin-1 elicits motor axon attraction, as indicated by axon turning towards the high end of the protein gradient [producing positive turning angles], at peak concentrations of 250 ng/mL and above (Figures 5A, B). Slit2, on the other hand, appeared to repel motor axons at peak concentrations of 100 ng/mL and above, although this effect did not quite reach statistical significance (Figures 5C, D). These results support the idea that floor plate Netrin-1 and Slits can elicit midline attraction and repulsion, respectively, in motor axons.

To determine whether Slits can directly silence, rather than just balance, the attractive effect of Netrin-1 on motor axons, we exposed motor neurons to a gradient of Netrin-1 (peak concentration of 250 ng/mL), either in the presence or absence of Slit2; here, Slit2 was not presented as a gradient but instead uniformly added to the media at 1 μg/mL, a concentration that likely far exceeds the threshold needed for Slit-mediated repulsion and should provide an excess of Slit even under full saturation of available Robo receptors. As expected, we again observed motor axon attraction to Netrin-1 (Figures 5E, F; Supplementary Figure S4) and found that bath-applied Slit2 on its own does not induce axon turning (Figures 5E, F; Supplementary Figure S4). Surprisingly, however, simultaneous exposure to a Netrin-1 gradient and evenly distributed Slit2 not only abolishes motor axon attraction to Netrin-1 but causes strong turning away from the source of Netrin-1 (Figures 5E, F; Supplementary Figure S4). Thus, Slit2 can convert Netrin-1’s attractive effect on motor axons to repulsion. This result is consistent with the idea that loss of Robo1 and Robo2 *in vivo*, while reducing or eliminating Slit-mediated midline repulsion, causes motor axons to gain attraction to floor plate-derived Netrin-1. It also suggests that wild-type motor axons might be repelled by Netrin-1 *in vivo*, as long as they are exposed to sufficient amounts of Slit proteins.

## Discussion

During development, spinal motor neurons extend axons to targets in the body periphery while their cell bodies remain anchored in the ventral horn of the spinal cord. A multiplicity of factors allows motor axons to leave the central nervous system, including mechanisms that prevent these axons from being attracted to inappropriate targets within the spinal cord (Suter and Jaworski, 2019). Previous work had demonstrated that genetic inactivation of the Slit receptors Robo1 and Robo2 in mice causes a subset of motor axons to remain within the central nervous system and extend across the floor plate at the ventral midline; similarly, motor neuron cell bodies leave the ventral horn and enter the commissure in *Robo1/2* double knockout mice (Bai et al., 2011; Kim et al., 2015; Kim et al., 2017; Gruner et al., 2019). To what extent these phenotypes are driven by loss of Slit-mediated repulsion from the floor plate or gain of responsiveness to the midline attractant Netrin-1 had remained unclear. We provide evidence that motor neuron entry into the midline is prevented by repulsion via Slit-Robo signaling and not influenced by DCC-mediated attraction to Netrin-1, whereas motor axon crossing of the ventral commissure in *Robo1/2* mutant mice results from an imbalance between Slit-mediated midline repulsion and Netrin-1 attraction. *In vitro* results indicate that Slits not only repel motor axons but can convert axonal responses to Netrin-1 from attraction to repulsion. These findings support the idea that Robo signaling allows motor axons to avoid the midline through two mechanisms: (1) by directly mediating repulsion from Slits and (2) by preventing DCC-mediated attraction to Netrin-1 and favoring repulsion from this cue.

### Robos prevent motor neuron midline entry, which is independent of DCC

Newly generated motor neurons migrate from their birthplace in the ventricular zone into the ventral horn, where they organize into functionally specialized columns and pools through adhesion-driven clustering (Demireva et al., 2011). Several mechanisms ensure that motor neuron cell bodies do not overshoot their settling position and follow their axons into the periphery, including inhibitory interactions with boundary cap cells (Vermeren et al., 2003), perineurial glia (Kucenas et al., 2008; Clark et al., 2014), and radial glia endfeet (Lee and Song, 2013) at MEPs, anchoring by the cell adhesion molecule TAG-1 (Suter et al., 2020), and signaling by secreted Semaphorins and Slits (Lee et al., 2015). Further, dorso-ventral positioning of motor neurons in the spinal cord is influenced by Slit-Robo and Netrin-DCC signaling, as they shift dorsally in *Netrin-1* and *DCC* mutant mice and ventrally in mice lacking Robo1 and Robo2 or all three Slits (Kim et al., 2015). Possibly as a consequence of these phenotypes, MEPs move dorsally or ventrally along with motor neuron cell bodies in these mutants, and these MEP shifts cancel each other out in *Netrin-1/Robo1/Robo2* triple mutants (Kim et al., 2017). Motor neuron positioning, however, in mice with simultaneous disruption of Robo and DCC signaling had not been directly assessed.

We focused on the ventral shifting of motor neurons in *Robo1/2* mutants, specifically the extreme case where motor neurons aberrantly enter the ventral midline. Consistent with previous work indicating that the severity of this phenotype declines with age (Kim et al., 2015), we find that it completely resolves itself between E10.5 and E11.5. We also show that motor neurons that enter the midline are exclusively of MMC identity, likely owing to their proximity to the floor plate, and that the total number of motor neurons in the spinal cord is comparable between wild-type and *Robo1/2* ^*−/−*^ mice. These results argue that, in the absence of Slit-Robo signaling, motor neurons are drawn into the midline by the floor plate, rather than being pushed by overcrowding in the ventral horn. As previously reported (Kim et al., 2015), the number of mispositioned neurons at E10.5 constitutes a very small fraction (<1%) of all motor neurons in the spinal cord. It remains unclear whether the birthdate, molecular profile, or migratory path into the ventral horn underlies the selective vulnerability of certain MMC neurons to aberrant midline entry. Interestingly, we find that motor neurons still enter the midline in *DCC* ^*−/−*^ *; Robo1/2* ^*−/−*^ mice*,* where Netrin-1-mediated floor plate attraction through DCC is abolished. This suggests that floor plate repulsion of motor neurons by Slit-Robo signaling prevents cell body entry into the midline by balancing other attractive signals from the floor plate. The alternative Netrin receptor DSCAM (Ly et al., 2008) might also contribute to motor neuron midline attraction, although the functional importance of this molecule for Netrin signaling in the mouse spinal cord is still unclear (Palmesino et al., 2012). It will also be interesting to understand the transient nature of the *Robo1/2* knockout phenotype, as it implies that motor neurons that enter the commissure undergo cell death, lose expression of motor neuron markers, or exit the commissure to join motor neurons on either side of the midline.

### Robo and DCC signaling have opposing effects on motor axon avoidance of the midline

In order to reach their peripheral targets, motor axons first need to leave the spinal cord via MEPs. Motor axons accomplish this feat by responding to peripherally expressed attractants, such as collagen XVIII (Schneider and Granato, 2006) and the chemokine CXCL12 (Lieberam et al., 2005). At the same time, they have to avoid navigating towards inappropriate targets within the central nervous system. Slits are produced by the floor plate and have previously been shown to act as repellants for motor axons *in vitro* (Brose et al., 1999), and deletion of *Robo1* and *Robo2* causes motor neurons to project axons across the midline (Bai et al., 2011; Kim et al., 2017; Gruner et al., 2019), supporting the idea that repulsion from the floor plate via Slit-Robo signaling is required to prevent motor axons from crossing the ventral commissure. It had remained unclear whether motor axon and cell body entry into the midline of *Robo1/2* knockout mice are interdependent and to what extent DCC-mediated attraction to Netrin-1 contributes to aberrant axon crossing of the midline in these mice.

We find that, similar to the cell body positioning defect, only a small subset (≈1-2%) of all motor neurons project axons across the midline in *Robo1/2* ^*−/−*^ mice; however, motor axons persist in the ventral commissure longer than motor neuron cell bodies, indicating that the axon guidance phenotype is not strictly dependent on neuronal mispositioning. The axonal defect does eventually resolve by E13.5, consistent with the idea that misprojecting axons are pruned. Through our analysis of *DCC* ^*−/−*^ *; Robo1/2* ^*−/−*^ triple knockout mice, we discovered that, at E10.5, DCC is a strong driver of aberrant motor axon midline crossing when Robo1/2 signaling is abolished, but this does not hold true at E11.5. These finding argue that, initially, Slit-Robo1/2 signaling is primarily required to counteract Netrin-1-dependent motor axon attraction to the floor plate, but, later on, Slit-mediated midline repulsion needs to balance out the attractive effects of other, yet-to-be-identified midline-derived factors. The possibly redundant contributions of Netrin-1 and other floor plate molecules to motor axon midline attraction at E11.5 remain to be determined, but increased responsiveness to these attractants might define the small subset of motor neurons that aberrantly project axons through the commissure in *Robo1/2* ^*−/−*^ mice. Of note, the partial requirement of DCC for motor axon, but not motor neuron, midline crossing in *Robo1/2* knockouts further underscores the independence of these two defects, and it suggests that Netrin-DCC signaling selectively attracts extending motor axons without influencing migrating cell bodies. This might indicate that DCC expression in motor neurons is low until they have settled in the ventral horn and begin to grow axons, or it could be explained by differential deployment and signaling activity of DCC in the axonal and cell body compartments.

### Crosstalk between Slit-Robo and Netrin-DCC signaling

The antagonistic relationship between DCC and Robo1/2 in motor axon midline crossing at E10.5 could indicate a balancing act between attraction and repulsion by floor plate-derived Netrin-1 and Slits, respectively; we will refer to this as the balancing model. However, it is also possible that Slit signaling suppresses motor axon attraction to Netrin-1 through Robo-DCC crosstalk, without playing a major direct role in midline repulsion, which we will refer to as the silencing model.

In line with published work (Brose et al., 1999), we found that Slit2 can repel motor axons. While function-blocking experiments using the Robo1 ectodomain *in vitro* had provided evidence against Slits being dominant drivers of motor axon repulsion from the floor plate (Patel et al., 2001), our E11.5 *in vivo* results, where DCC does not promote motor axon midline crossing and the silencing model is excluded, are most readily explained by Robo-dependent motor axon repulsion from midline-derived Slits. This apparent discrepancy might be due to incomplete blocking of Slit activity *in vitro* or higher sensitivity of motor axons to Slits *in vivo*. No matter the explanation, the balancing model, which requires Slit-dependent midline repulsion, could therefore also apply at E10.5. Nonetheless, prior evidence for the silencing model is strong, and our data provide further support. DCC and Robos physically interact, and genetic deletion of the γ-secretase component Presenilin-1 (PS1), which leads to accumulation of intracellular DCC “stubs” that cannot bind Robos and are thought to circumvent silencing, causes motor axon midline crossing; this phenotype is rescued when *DCC* is knocked out, indicating that DCC drives ectopic midline crossing in mice lacking PS1 (Bai et al., 2011). For the silencing model to remain viable, the *Robo1/2* knockout phenotype had to be similarly DCC-dependent, and this is exactly what we find, at least at E10.5. Further, motor neuron explant experiments at early developmental stages had shown that motor axons fail to grow towards Netrin-1-expressing cells (Varela-Echavarría et al., 1997), but blocking Slit signaling with the Robo1 ectodomain in ventral spinal cord explants allows motor axon attraction by Netrin-1 (Bai et al., 2011). This is readily explained by the fact that motor neurons themselves secrete Slits for autocrine signaling (Jaworski and Tessier-Lavigne, 2012), and motor neuron- and floor plate-derived Slits could therefore both contribute to Netrin silencing. In our axon turning assays, due to the absence of floor plate and the low density of neurons, endogenous Slit levels are likely to be very low, explaining why we observe robust attraction to Netrin-1. This allowed us to directly test whether addition of Slits changes motor axon responses to Netrin, and we found that high concentrations of Slit2 convert Netrin-1 attraction to repulsion. This is the inverse of the Netrin-Slit crosstalk observed in thalamocortical axons, where Netrin-1 can convert repulsive effects of Slit1 to attraction (Bielle et al., 2011). It is possible that, *in vivo*, motor axons respond to Netrin-1 on a continuum that ranges from attraction to repulsion, depending on the level of Slits they are experiencing at any given moment; the range of Slit concentrations that can flip the valence of Netrin chemotactic signaling, as well as the local, physiologically relevant Slit concentrations that extending motor axons are exposed to *in vivo*, remain to be determined. While the mechanism of the observed Slit-Netrin crosstalk remains elusive, it could involve direct binding between the ligands (Brose et al., 1999) and/or their receptors (Bai et al., 2011), or the intersection of downstream signaling pathways. Irrespective of the molecular mechanism, our data are consistent with the idea that Slit-Robo signaling suppresses, or even inverts, attractive motor axon responses to midline-derived Netrin-1, which is a variation of the silencing model. At E10.5, this mechanism might act alone or in parallel to Robo-mediated repulsion from midline-derived Slits to help motor axons steer clear of the floor plate.

At E11.5, DCC is no longer a major contributor to aberrant motor axon midline crossing in *Robo1/2* mutant mice. While this strongly argues for Slit-dependent repulsion becoming the predominant mechanism for Robo function in this context, it also raises the question why DCC silencing by Robos, provided it operates at E10.5, is less important at this age. Interestingly, an intracellular p190RhoGAP-dependent mechanism for inhibiting motor axon attraction to Netrin-1 has been found to prevent motor axon misrouting along the pial surface of the spinal cord (Bonanomi et al., 2019). While disruption of this pathway alone does not cause motor axon midline crossing (Bonanomi et al., 2019), it remains possible that it helps dampen attraction to floor plate-derived Netrin-1, partially relieving Robo1 and Robo2 of this responsibility. Ultimately, our data support roles for Robos in both Slit-mediated midline repulsion and the modulation of Netrin-1 responses in motor axon avoidance of the midline, but the precise relative contributions of these mechanisms, as well as other Netrin silencing mechanisms, at different developmental stages remain to be resolved.

## Data Availability

The original contributions presented in the study are included in the article/Supplementary Material, further inquiries can be directed to the corresponding author.

## Supplementary material

SUPPLEMENTARY FIGURE S1 Total number of motor neurons in E10.5 wild-type and Robo1/2^−/−^ mice. The total number of motor neurons per hemisection was quantified. There is no significant difference between E10.5 wild-type and Robo1/2^−/−^ mice (n = 5–8 animals/group, p = 0.0723). Data are represented as means ± SEM.

SUPPLEMENTARY FIGURE S2 Mispositioned motor neurons are not observed beyond E10.5. **(A)** E11.5 and E13.5 wild-type, DCC^−/−^, Robo1/2^−/−^, and DCC^−/−^; Robo1/2^−/−^ mouse spinal cord sections were stained for Isl1/2 and Tuj1. Isolated Isl1/2 channel is shown in greyscale. **(B)** Quantification of mispositioned motor neurons in E11.5 and E13.5 wild-type, DCC^−/−^, Robo1/2^−/−^, and DCC^−/−^; Robo1/2^−/−^ mice shows no differences in any mutant genotype compared to age-matched wild type. Data are represented as means ± SEM (n = 3–5 animals/group). Scale bar = 100 µm.

SUPPLEMENTARY FIGURE S3 Motor axons do not aberrantly cross the midline at E13.5. **(A)** E13.5 wild-type, *DCC* ^*−/−*^, *Robo1/2* ^*−/−*^, and *DCC* ^*−/−*^ *; Robo1/2* ^*−/−*^ sections were stained for NF and Prph. **(B)** Quantification of Prph^+^ motor axons crossing the midline showed that the previously shown phenotype (Figure 3) is not observed in any mutant genotype or wild type at E13.5. Data are represented as means ± SEM (n = 3–4 animals/group). Scale bar = 100 µm.

SUPPLEMENTARY FIGURE S4 Distribution of individual axon turning angles in Dunn chambers. Quantification of individual axon turning angles measured in Dunn chambers shows spread of data points through attraction (positive angles) and repulsion (negative angles). Data points are color-coded, identifying origin of individual experiments for the conditions. Data are represented as means ± SEM.
