# Supplementary figures and images for: Slit-Robo signaling supports motor neuron avoidance of the spinal cord midline through DCC antagonism and other mechanisms
Source: Front Cell Dev Biol. 2025 Apr 10;13:1563403. doi: 10.3389/fcell.2025.1563403 (PMC12018395; doi:10.3389/fcell.2025.1563403)

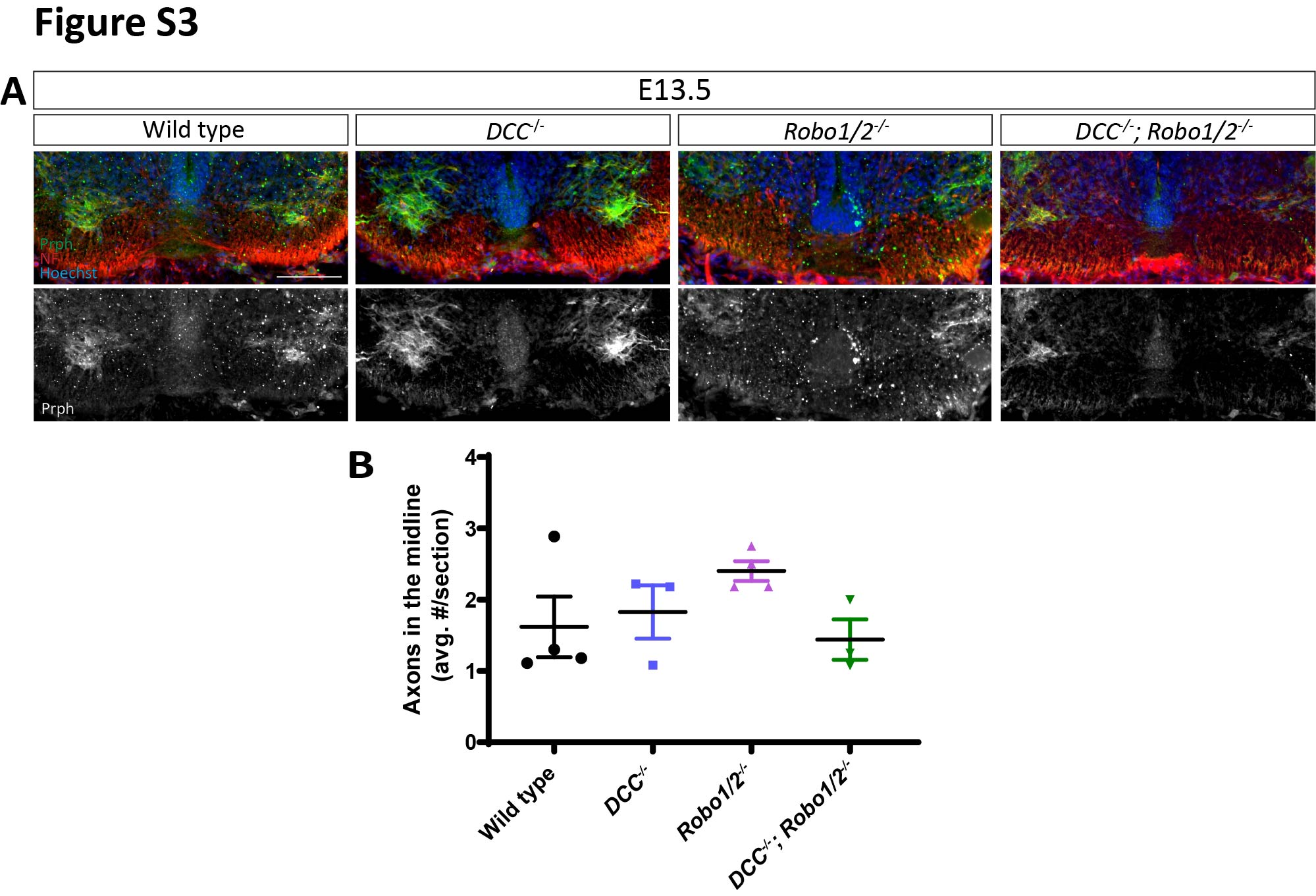

Supplement: Supplementary file 1 [file Image3.jpeg]

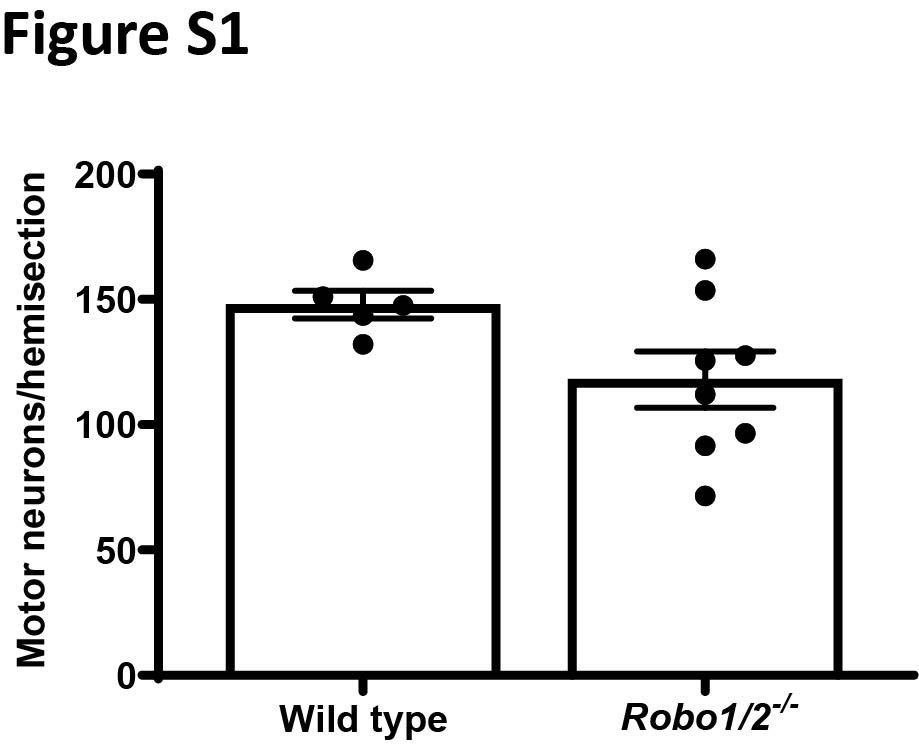

Supplement: Supplementary file 2 [file Image1.jpeg]

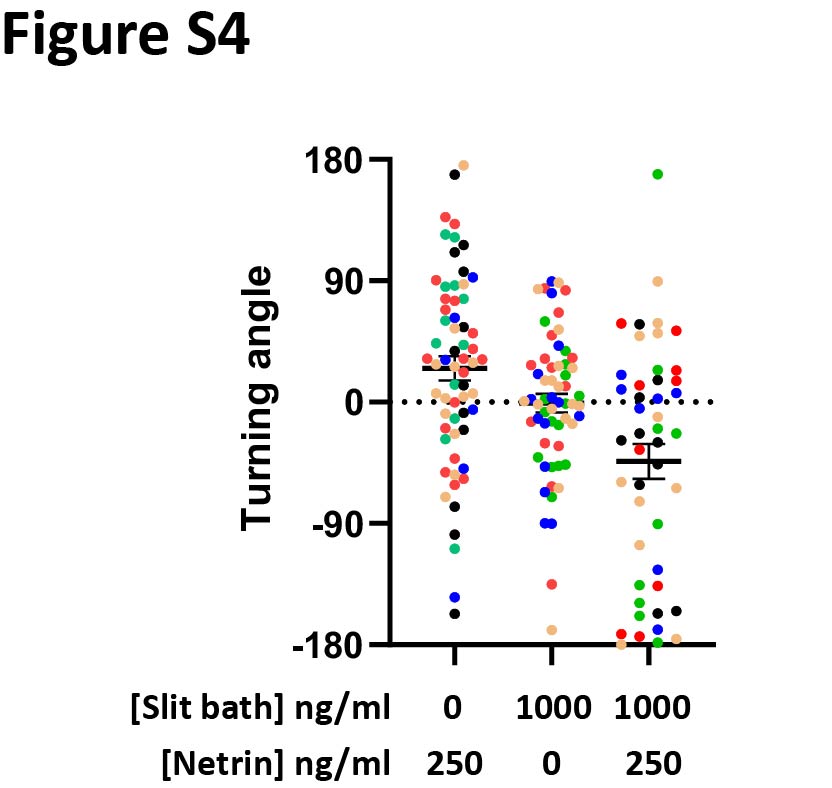

Supplement: Supplementary file 3 [file Image4.jpeg]

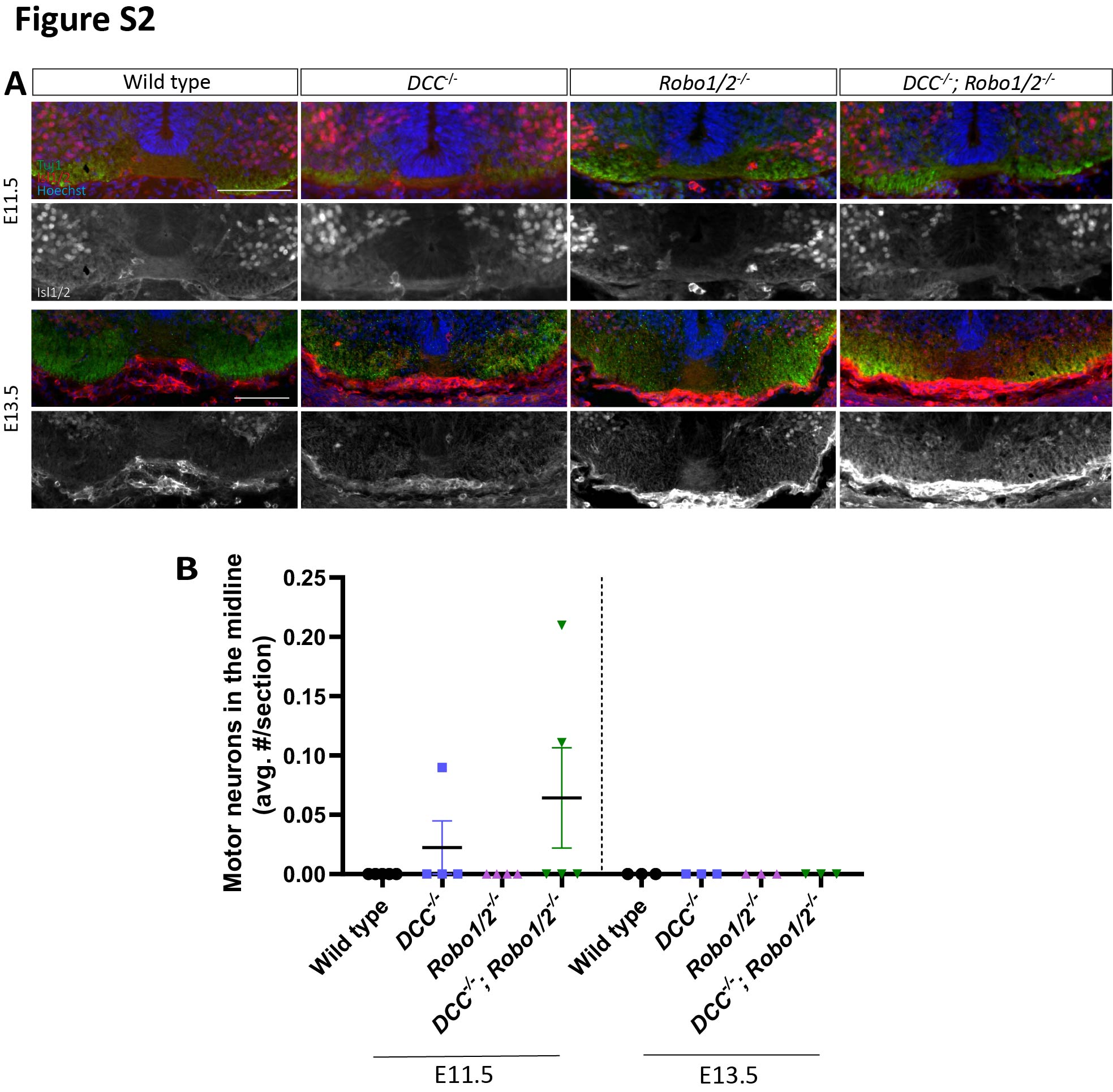

Supplement: Supplementary file 4 [file Image2.jpeg]
